# Applied Pediatric Integrative Medicine: What We Can Learn from the Ancient Teachings of Sebastian Kneipp in a Kindergarten Setting

**DOI:** 10.3390/children5080102

**Published:** 2018-07-26

**Authors:** Marion Eckert, Melanie Anheyer

**Affiliations:** 1 Kinderkrankenhaus St. Marien, 84036 Landshut, Germany; 2 Elisabeth Krankenhaus, 45138 Essen, Germany; m.anheyer@contilia.de

**Keywords:** Kneipp, kindergarten, PIMR

## Abstract

Pediatric integrative medicine focuses on the whole child and the environment in which the child grows up during the treatment of a child’s illness. Nowadays, many different treatment modalities are applied even in children, and doctors need to know about them and, ideally, be able to apply different approaches in the process of treating a child themselves. The program Pediatric Integrative Medicine in Residency (PIMR) already provides residents with several tools to provide this kind of service for the child. In our PIMR pilot program in Germany, we chose to diversify our knowledge about treatment and prevention options by visiting a Kneipp-certified kindergarten in Germany. The philosophy of Sebastian Kneipp focuses on five pillars of health, which incorporate aspects of prevention, self-awareness, self-responsibility, and consciousness of health by means of hydrotherapy, herbal medicine, exercise, nutrition, and lifestyle-medicine. These are being taught to the children during the early years they spend in kindergarten, and represent integral parts of integrative medicine. Integration of Kneipp-based health programs within a kindergarten setting can work well and provides an effective means of early prevention education in childhood.

## 1. Introduction

In Germany, there has been a long tradition of naturopathic medicine. Hildegard von Bingen (1098–1179), a Benedictine abbess, is considered the founder of natural history and her work still greatly influences modern naturopathic medicine movements. One of the more recent forefathers of the naturopathic movement was the priest, Sebastian Kneipp (1821–1897), who is most commonly associated with hydrotherapy and the “Kneipp-Cure”, which he claimed had therapeutic or healing effects. But, it was not only hydrotherapy that he based his principles of achieving and maintaining health on. In fact, he proposed an entire system of healing, which rested on five pillars: Hydrotherapy, herbal medicine, exercise, nutrition, and lifestyle-medicine (order and balance) [1]. Even nowadays, his teachings and philosophy are a part of many naturopathic teachings in Germany, being represented in national and regional Kneipp-associations who certify a wide variety of qualified facilities with the “Kneipp certificate”, such as schools and kindergartens as well as health resorts and spas, campgrounds, and guest houses, who must fulfill certain requirements according to the Kneipp philosophy. As Kneipp’s principles form the basis for European traditional naturopathic medicine, they are an essential part of the curriculum offered for physicians in Germany who want to become legally certified naturopathic doctors. The pillars of Kneipp can also be found in modern and international integrative and naturopathic medicine and teaching curriculums [2,3], which makes them an essential part of knowledge for the integrative physician.

Integrative medicine in pediatrics focuses on the whole child and the environment in which the child grows up. Currently, obesity in children and allergies as well as stress related disorders are on the rise. An immediate reward principle, with everything readily available and a focus outside of oneself, is an increasing phenomenon. Therefore, prevention aspects, as well as self-awareness and mindfulness in life and health, have become important aspects of modern medicine. Especially, children need tools to grow up healthy in this kind of environment. Nowadays, many treatment modalities are being applied in integrative medicine and in the field of pediatrics [2]. The teaching program, “Pediatric Integrative Medicine in Residency (PIMR)” [3], provides residents with the knowledge about a wide variety of complementary healing approaches. In Germany, the PIMR program was used as a pilot program at three different childrens’ hospitals. To deepen and diversify their knowledge about integrative medicine approaches, the group explored the principles of a Kneipp-certified kindergarten in Germany as an extracurricular activity. As described above, the philosophy of Sebastian Kneipp focuses on five pillars of health, which incorporate prevention, self-awareness, self-responsibility, and consciousness of health. These principles are being taught to the children during the years they spend in kindergarten.

## 2. Visit of the Kneipp Kindergarten

### 2.1. General Information

The kindergarten, St. Stephanus, in Essen, Germany, is one of 400 Kneipp-certified child daycare facilities in Germany. Certification by the Kneipp-Bund requires the offer of a multitude of self-awareness and self-experience opportunities that lie within the Kneipp principles to attain the ability of prevention in everyday life. In the case of a kindergarten, at least 50% of the teachers are required to attend an advanced 30-h training in health concepts according to Kneipp, and must attend yearly refresher courses. For implementation in daily life, a set of guidelines has been laid out, which must be fulfilled by the kindergarten to be certified [4]. These guidelines incorporate adequate outdoor and physical activity spaces, integration of parents, balance of life (e.g., daily routine, time for sleep and rest), offer of physical activities in- and outdoors, healthy nutrition, use of herbs, and, finally, daily water treatments according to Kneipp. After 18 months of gaining experience, certification of the facility ensues.

In the facility of St. Stephanus, the children are divided into three groups, with a total of 54 children. Two of the groups have children aged 3–6 years and one group is for children aged 0.3 months–3 years of age. The children can stay in the kindergarten either 35 h (7:00 a.m. through 2:00 p.m.) or 45 h (7:00 a.m. through 4:30 p.m.) per week. Still, there are many intergroup activities so children experience a greater variety of experiences and decision processes. There is a total of six teachers, one pediatric nurse, one childcare assistant, and the director of the facility. Children have two group rooms, two side rooms, a big common room, two wash rooms, a sleep room, one activity room, and a wide outdoor area at their disposal. They can play in all weather conditions, as the facility considers itself an all-weather kindergarten.

This kindergarten is closely associated with the local catholic community. Religious work is characterized by the appreciation of others and their beliefs, so acceptance and tolerance of different religions is actively lived. St. Stephanus is under the administration union of the “KiTa Zweckverband”, a union of child daycare facilities in the Bishopric of Essen, and is financed partly by the union, the parents, and the city of Essen.

St. Stephanus is situated amidst one of the most densely populated parts of the city of Essen, namely Essen Holsterhausen, with close to 27,000 inhabitants. This part of Essen is characterized by a mixed population of all social classes where many single parents are in need of long hours of care for their child. The kindergarten is well accessible by car, bus, and is in walking distance of the surrounding residential areas.

### 2.2. Conceptual Design

Life in the kindergarten, St. Stephanus, has been lived according to the principles of Sebastian Kneipp since April 2013, when the kindergarten was officially certified by the “Kneipp-Bund”.

As soon as you enter the facility the spirit of Kneipp’s principles becomes evident: Binders in different colours and decorated stones representing the five pillars welcome the visitor. Throughout the kindergarten, it is obvious how deeply the Kneipp concept is integrated into everyday life. The following examples show how each of the five pillars of hydrotherapy, phytotherapy, physical activity, nutrition, and lifestyle-medicine is lived and applied by the children and their teachers.

Hydrotherapy is applied in many ways: The children learn through the effects of cold- or warm water washings or brushings how to calm themselves down or energize themselves. Every child has their own washcloth or brush and learns how to use it. There are tubs for arm-baths and foot-baths. In winter, children with warm feet go for a short walk in the snow, and, in spring and summer, the dew on the lawn is used for the “dew cure”. This is one of the more than 120 forms of hydrotherapies of Kneipp, where a barefoot walk with initially warm feet in the fresh morning grass is performed for 2–5 min to boost the circulation, and strengthen the immune system and foot muscles. Water basins with cold water are used for water treading, the most well-known water application of Sebastian Kneipp. Always, the individual child, its health condition, body temperature etc. is considered so the child learns to listen to signs of their own body to determine what is good for them and what can be potentially harmful. Therefore, a self-determined and responsible care for their own health and body condition is taught to the children.

Herbs also play a central role in the kindergarten. An outdoor herb garden is used for planting and identifying herbs with the children. They pick and dry them and use them for making herb sachets and herbal teas as well as reference books. Also, aroma-oil diffusions are done in the group rooms, and herbal inhalations in a tent under supervision of a teacher. This way the children learn about the properties of the herbs and how they can be applied. Herbs used are common traditional European medicinal plants, for example chamomile, fennel, peppermint, anise, and lavender. Throughout the kindergarten, herbs and information sheets on how to use them are displayed for parents. This provides the families and children with respect and knowledge about nature as well as with tools for their own health.

Physical activity is also an important module in the kindergarten as well as promoting feeling and sensing the own body. Hence, there is a movement room in which children can move freely, climb, and be wild. To promote sensation, all is done barefoot. Also, the large outdoor area of the kindergarten gives the children the opportunity to move around outdoors in all weather conditions. With a feeling parcours, on which they also walk barefoot, children learn the different feeling of grass, asphalt, rocks, snow etc. in a mindful way. Thus, the children’s innate joy of movement can be considered. The aim is to promote this in a sustainable way, which also shapes the social and emotional development of the children.

In early childhood, the foundations for a consciousness of healthy nutrition and way of eating can be built and shaped. Nutrition in the kindergarten is according to the recommendations of the Germany society for nutrition, incorporating seasonal and regional foods as well as whole grain products. A healthy diet is promoted through a daily changing breakfast buffet from which children can choose, serve themselves, and thereby determine what it is they want to eat. The lunch menu is selected each week by a different group of children. This way the children learn to listen to their body’s needs while being able to choose from a healthy buffet.

The principle of order and balance is taught by the rules of Papilio for children as well as through daily and seasonal rituals, which promote a feeling of safety and assurance for the children. Balance between sleep and activity during the day, and a regulated day with fixed meal and play times is as important as lunch rituals, sleep rituals, birthday rituals, etc. Papilio is a program for the primary prevention of behavioral problems and for supporting the socio-emotional competencies of children. It has been designed for three different age groups and aims for the prevention of addictions and violence. The program is based on scientific studies and the effects have been proven by a controlled longitudinal study [5]. The kindergarten uses the program designed for children aged 3–6 years. According to the principles of Papilio, children learn how to interact with themselves and others in a respectful way, how to contribute their own matters, and how to be considerate of others.

The first principle is: Learn how to play with others and how to make yourself happy without the use of specific toys. Therefore, one day per week there is a “toys are on vacation day” where children only work with natural materials or must come up with own interactive ideas for playing.

The second principle is learning social rules and awareness of others in a playful way. Daily rules are made up together with the children, and one child always makes sure the rules are kept. For example, “I let others finish their sentence”, “I listen to what others are saying”, and “I put toys away after I’m finished playing with them”.

The third principle is about teaching the children about emotions, so that they learn to recognize these in themselves and others, and, this way, learn how to deal with them. Puppets, in the form of emotion-goblins, displaying the major emotions of anger, happiness, sadness, and fear are applied in various ways. The happiness-goblin always smiles and cannot understand that anyone could feel different, the anger-goblin could explode upon every opportunity, the crying-goblin is always sad, and the fright-goblin is always afraid because something bad could happen at any time. The children learn how to recognize their own feelings as well as the feelings of others, and develop an understanding for the feelings of someone else. This is an important task in the development of a personality.

## 3. Discussion

In the kindergarten of St. Stephanus, the children live the principles of Kneipp in everyday life. In a playful way, they learn the basics for a healthy lifestyle, not only about “what can make me sick”, but, even more so, “what keeps me healthy”. Health-conscious behaviors and attitudes are being learned and practiced so that they become natural to the child and can provide the basis for a better consciousness of health and a healthier lifestyle later in life.

Despite health problems being a rising phenomenon in children, health education is usually not a teaching subject in schools and kindergartens. Different concepts of education can be found in kindergartens in Germany, yet, a concept based on physical and mental health education is unique to a Kneipp kindergarten.

The principle of order and balance forms the basis for the other four pillars according to the principles of Kneipp. It helps and supports children in implementing health-promoting attitudes into their daily life. In the kindergarten, this is achieved by the rules according to Papilio, as well as rituals, so that children can orient themselves and have a sense of stability. Thus, they can acquire skills that help them to find the balance between resources and everyday requirements.

There are no studies that have been performed specifically on Kneipp kindergarten children. However, studies done on children in various ages and adults show that water treatments lead to active training of blood vessels. Cold and warm water treatments increase blood flow and result in the training of blood vessels, which leads to a stabilization of the immune system [6]. A variety of benefits have been described, including a lowering of the frequency of coughing in respiratory tract infections and various immunological modulations [7,8,9,10,11]. As most children love water, washings, arm- and foot-baths, and walking in dew, snow, and water are perfect ways to playfully help in the prevention of common colds [7]. By directly experiencing their bodily reactions to the water-stimuli, they can learn to develop a better awareness of their own body.

Herbs, which the children grow in their own herb garden, are an essential component of complementary medicine as well as an integral part of the Kneipp philosophy. The direct contact with nature teaches the children respect and lays the foundation of an understanding of the benefits and damages of nature. The child recognizes the involvement of the human being in its environment. The sense of responsibility towards the environment and one’s own body is supported and promoted.

Physical activity is a fundamental need and an integral part in the mental and physical development and well-being of children. In the kindergarten of St. Stephanus, the children are allowed to follow their daily urge to move. The focus is on elementary forms of movement and combines movement with positive experiences. This can prevent physical inactivity, which is associated with various diseases, such as metabolic syndrome and back pain.

Sebastian Kneipp recognized that many diseases occur due to the wrong type of nutrition. Therefore, he promoted a simple, natural diet with enough calories to support the functions of the organs and the body. Nutrition has also gained importance in conventional medicine, as the influence of malnutrition and over-nutrition on the development of civilization diseases is widely recognized nowadays. In St. Stephanus, the children receive a healthy, balanced diet, which is as natural and seasonal as possible. This can help establish a healthy eating pattern in the children. As parents are integrated into the activities of the kindergarten, this is also transported into the homes and families of the children.

Many publications show that despite huge medical advances, many children suffer from lifestyle (obesity, stress related disorders), chronic, and severe diseases [12,13,14,15]. Therefore, the promotion of health and wellness in childhood is essential. Kneipp had already recognized, in the 19th century, that the relationship between the body, mind, and soul plays a pivotal role in health and disease. Accordingly, he placed the whole human being, with its physical, psychological, and social needs, in the center of his prevention and therapy system, just like modern integrative medicine is dedicated to. He demanded responsibility for one’s own health, including an active participation in one’s own lifestyle and social relationships. Apart from a certain way and order of life, he claimed that physical activity, nutrition, and the use of herbs can be used to prevent and treat sickness. Those aspects are an integral part of modern integrative and complementary medicine, and are also represented in the curriculum of the PIMR [3].

The fact that this can already effectively be applied to children makes the Kneipp concept an ideal target for kindergartens and early priming for a healthy lifestyle. Most children are open and curious and love to learn [16,17]. This provides an excellent ground for laying new and lasting foundations regarding their own health. Teaching them Kneipp’s prevention and treatment tools in a fun way at an early age provides an effective way to make those principles a palpable experience for children and allows for a holistic childhood education.

The limitation of applying some of Kneipp’s principles in a kindergarten lies in the fact that treatments of sick children cannot be performed by teachers or nurses due to legal issues. Therefore, the use of Kneipp is limited to prevention aspects in this setting.

In Germany, no curriculum for a certification in naturopathic or integrative pediatrics exists. To obtain the certification of a naturopathic physician, only doctors can go through a curriculum designed for adult patients. In this curriculum, the principles according to Kneipp are an essential part and even a specific certification as a Kneipp physician exists. Visiting a Kneipp certified kindergarten helps pediatric residents and physicians to directly gain knowledge about the application of those principles to children so that they can use that knowledge in their care of children and transfer theory into practice. In this way, they can advise parents or educate kindergartens and schools in the application of Kneipp’s principles and, therefore, support the inclusion of health promoting activities (both physical and social-emotional) in educational settings. Also, certain modalities, like hydrotherapy, can be introduced by adept doctors into pediatric hospitals interested in holistic healthcare approaches.

## 4. Conclusions

It has been shown that even in early childhood basic attitudes and patterns regarding one’s own health and healing can be developed. Kneipp’s system of healing, with its pillars of hydrotherapy, phytotherapy, exercise, and nutrition as well as order and balance, can be well integrated within a kindergarten setting and are perfectly suitable for teaching children about responsibility and connectedness towards self, others, and nature. In this way, the Kneipp principles can provide holistic support for the development of personality and health-conscious behaviors of the child. An awareness of individual health resources and avoidance of risk factors are promoted, as well as the development of a natural feeling for physical and emotional health, respect for others and the environment, mindfulness towards one’s own resources, self-empowerment, and self-responsibility for health and emotional competencies.

A significant proportion of children worldwide suffer from health problems and with an increasing age the risk of civilizational diseases rises. A healthy lifestyle can prevent diseases or diminish their progression. Child day care facilities offer an ideal setting to introduce children to a health-promoting and health-conscious way of living: For one, children are curious, and their behavioral patterns can still be shaped. Secondly, kindergarten is a universal area of life for pre-school children, which makes it possible to reach children and parents from all social backgrounds. Even though there are currently no studies existing that give evidence about the advantages of a Kneipp based education in kindergarten, the principles of Kneipp are integral parts of modern integrative and naturopathic medicine. Kneipp based educational systems for children provide a good opportunity for physicians to observe and promote self-care strategies applied in children.
